# Multi‐surveyor capture‐mark‐recapture as a powerful tool for butterfly population monitoring in the pre‐imaginal stage

**DOI:** 10.1002/ece3.9140

**Published:** 2022-07-31

**Authors:** Heiko Hinneberg, Jörg Döring, Gabriel Hermann, Gregor Markl, Jennifer Theobald, Ines Aust, Thomas Bamann, Ralf Bertscheit, Daniela Budach, Jana Niedermayer, Alicia Rissi, Thomas K. Gottschalk

**Affiliations:** ^1^ University of Applied Forest Sciences Rottenburg Rottenburg am Neckar Germany; ^2^ Private Laupheim Germany; ^3^ Arbeitsgruppe für Tierökologie und Planung GmbH (Filderstadt) Filderstadt Germany; ^4^ University of Tübingen, Petrology and Mineral Resources Tübingen Germany; ^5^ Regierungspräsidium Tübingen, Referat 56 ‐ Naturschutz und Landschaftspflege Tübingen Germany; ^6^ Private Tübingen Germany; ^7^ ISTE Baden‐Württemberg e. V. Ostfildern Germany; ^8^ Institut für Landschaftsökologie und Naturschutz (ILN) Bühl Bühl Germany

**Keywords:** caterpillar, detection probability, Lepidoptera, *Limenitis*, overwinter mortality, population size, sessile life stages

## Abstract

For many elusive insect species, which are difficult to cover by standard monitoring schemes, innovative survey methods are needed to gain robust data on abundance and population trends. We suggest a monitoring of overwintering larvae for the endangered nymphalid butterfly *Limenitis reducta*. We tested different removal and capture‐mark‐recapture (CMR) approaches in a field study in the “Alb‐Donau” region, Germany. Classical removal and CMR studies require movement of the organisms under study, but in our approach, we replaced movement of the study organisms by random movement of multiple different surveyors. We tested the validity of the approach by comparing detection frequencies from our field data with simulated detections. Our results indicate that multi‐surveyor removal/CMR techniques are suitable for estimating abundance of overwintering *L. reducta* larvae. Depending on surveyor experience, the average detection probability ranged between 16% for novices and 35% for experts. The uncertainty of population estimates increased with a decrease in personnel expenditure. Estimated larval densities on a spruce clear‐cut varied between one and three individuals per 100 m^2^, probably related to habitat conditions. We suggest a CMR approach with three to four trained surveyors for the monitoring of *L. reducta* populations in the overwintering stage. Compared with previous sampling methods, our approach is a powerful tool with clear advantages: long survey period, estimates of the absolute population size accompanied by uncertainty measures, and estimates of overwinter mortality. The proposed method can be adapted and used for several different butterfly species, other insect taxa with specific immobile life stages, and some sessile organisms, for example, elusive plants, fungi, or corals.

## Graphical abstract

Testing removal and capture‐mark‐recapture approaches within a population of overwintering caterpillars of the nymphalid butterfly Limenitis reducta, we showed that the quantitative study of pre‐imaginal butterfly life‐stages can provide reliable abundance data, useful for monitoring and comparative studies. The innovative aspect of our method is that we substituted mobility of the study organism, which is a prerequisite in classical capture‐mark‐recapture studies, by multiple surveyors sampling independently of one another. We believe that this approach can be easily applied and fitted to other species, for example, insects with specific sessile life stages, plants, fungi, and corals.

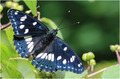

## 1 INTRODUCTION

A growing body of evidence from different geographical regions records the decline of terrestrial insects in general (Eggleton, 2020; Van Klink et al., 2020; Wagner, 2020) and of Lepidoptera in particular (Costache et al., 2021; Forister et al., 2011; Franzén & Johannesson, 2007; Habel et al., 2019; Wepprich et al., 2019). Butterflies are among the best‐studied insect families, and butterfly monitoring schemes have been established in many countries during the last decades (e.g., Feldmann et al., 2005; Henry et al., 2005; Stefanescu et al., 2011; Van Swaay et al., 1997, 2008), providing great potential for detecting population trends. Monitoring of butterflies is usually conducted as transect walks with counts of imagines (Pollard, 1975). On the one hand, this method is well‐suited for deriving population indices for many common species but it has some shortcomings on the other hand. For example, current survey schemes fail to provide reliable information for cryptic or low‐density species (Gottschalk, 2020), they estimate an index of species abundance instead of the absolute abundance in a specific area, and the uncertainty of individual population indices cannot be quantified (cf. Nowicki, 2017; Seber, 1982).

So far, the possibilities that monitoring of butterflies in pre‐imaginal stages provide were often ignored. But putting the study of pre‐imaginal butterfly stages forward appears very promising due to several reasons. (1) ecological reasons: Pre‐imaginal stages, that is, eggs and larvae, are particularly susceptible to stress factors caused by environmental changes (Mulé et al., 2017; Radchuk et al., 2013), but they often have no or limited dispersal capacities to avoid or reduce this stress (e.g., Weiss & Murphy, 1988). The abundance of pre‐imaginal stages is, therefore, probably more directly linked to local habitat quality than the abundance of adults (Dempster, 1997). Hence, when trying to relate changes in species abundance to changes of the environment, all life stages have to be considered with a special emphasis on pre‐imaginal stages (Radchuk et al., 2013). (2) methodological reasons: Monitoring butterflies in the pre‐imaginal stage, and especially during hibernation, allows to overcome the problem of temporal population fragmentation, resulting from the often short life span of individual adult butterflies relative to the length of the flight period (Bubová et al., 2016; Nowicki et al., 2008). Only a rather small fraction of adults belonging to a single generation occurs and can be recorded at any moment of time. Consequently, adult counts cannot be directly extrapolated to seasonal population sizes. In contrast, in the case of larvae or eggs, all individuals of the same generation are present together at least for some periods. Monitoring of pre‐imaginal stages can facilitate survey planning because pre‐imaginal stages represent the longest part of the life cycle in many European butterflies (Bubová et al., 2016; Fartmann & Hermann, 2006; Settele et al., 2015) and, in contrast to adults, for many species their detectability is not strongly dependent on weather conditions. Furthermore, pre‐imaginal stages always occur in higher numbers compared with adults, increasing the number of detections in some species and providing sufficient sample sizes for statistical analyses (Hermann, 2007).

Egg counts and records of larval webs play an important role in the study of habitat requirements and oviposition preferences (Anthes et al., 2003; Pennekamp et al., 2013; Pschera & Warren, 2018). However, they were only used in some flagship species such as *Euphydryas maturna*, *Melitaea cinxia*, *Phengaris alcon*, and the lappet moth *Eriogaster catax* to provide information on species abundance (Dolek et al., 2018; Hanski, 2011; Hanski & Singer, 2001; Kajzer‐Bonk & Nowicki, 2022; Nowicki, 2017; Ojanen et al., 2013). Besides some knowledge gaps concerning species‐specific host plant preferences, determining the detection probability of different observers is the main difficulty preventing the use of egg counts and larval records for abundance estimation in a larger number of species. One approach to determine the observer‐specific detection probability might be through repeated sampling within the same population, that is, through removal or capture‐mark‐recapture (CMR) experiments. These approaches explicitly estimate detection probability and, therefore, allow the quantification of species abundance (Birch et al., 2021; Haddad et al., 2008; Rodriguez de Rivera & McCrea, 2021). Removal and CMR appear promising for the quantitative study of pre‐imaginal butterfly stages, although both approaches have been largely ignored in this regard, so far (but see Weseloh, 1985).

When animals are trapped with a constant sampling effort and removed from the study region over multiple occasions, the number of captured individuals will likely decrease in later capture occasions as long as no birth nor immigration into the population occurs. The rate of decrease in the number of captured individuals can provide an estimate for the size of a population, known as the “removal method” (Leslie & Davis, 1939; Moran, 1951; Rodriguez de Rivera & McCrea, 2021; Zippin, 1958). Estimating animal abundance with the removal method is particularly suitable in cases where individuals are routinely removed from a study region, for example, fisheries or the management of unwanted/invasive species (Cowx, 1983; Davis et al., 2016; Leslie & Davis, 1939; Yuksel et al., 2013; but see Schori et al., 2020). When, however, the conservation of rare species requires precise estimates of population sizes, CMR techniques are more commonly applied (e.g., Dolek & Geyer, 2000; Jackson et al., 2006; Kadlec et al., 2010; Pennekamp et al., 2014). Population estimates from CMR or removal approaches can reach a high accuracy and—unlike counts—are typically accompanied by a measure of uncertainty. Closed population capture‐mark‐recapture models (i.e., without removal), first described by Petersen (1896)/Lincoln (1930) and Schnabel (1938), are among the most fundamental approaches for estimating animal abundance. Like removal models, closed population CMR is based on three important assumptions: (1) The absence of births and deaths (demographic closure), (2) no movements across the borders of the study area (geographic closure), and (3) equal catchability of all individuals (Conroy & Carroll, 2009; Rodriguez de Rivera & McCrea, 2021). Closed population CMR surveys include at least two different occasions of species recording. During the first occasion, individuals are marked and subsequently released back into the population. In the second and any following occasion, the number of marked and unmarked individuals in the sample is recorded and further individuals may be marked.

The assumption of equal catchability does usually require mixing of marked and unmarked individuals after the first capture occasion in a CMR experiment and, therefore, movement of the organism under study. However, we propose that it is conceptually possible to substitute random mixing of individuals by random search patterns during different occasions of species recording. This makes removal and CMR approaches applicable to butterflies in pre‐imaginal life stages and other sessile organisms. To realize random search patterns in the field, we suggest replacing the surveyor after each survey by a new and uninformed surveyor.

We conducted a field study with multiple independent surveyors in a population of overwintering larvae of the endangered Southern White Admiral (*Limenitis reducta* Staudinger, 1901, Figure 1). Our study consisted of detections/redetections without physical capture or removal of individuals. However, in the analysis of our data, we applied principles analogous to those of classical removal/CMR studies and we therefore use removal/CMR terminology. The aims of our study were to test the applicability of removal and CMR approaches for estimating population size of hibernating butterfly larvae, to derive density estimates for *L. reducta* larvae under different habitat conditions, and to suggest a scheme for future population studies of this highly endangered species. We discuss the strengths and weaknesses of the proposed methodology and how it can contribute to improve the quantitative study of butterflies and other immobile organisms.

**FIGURE 1:**
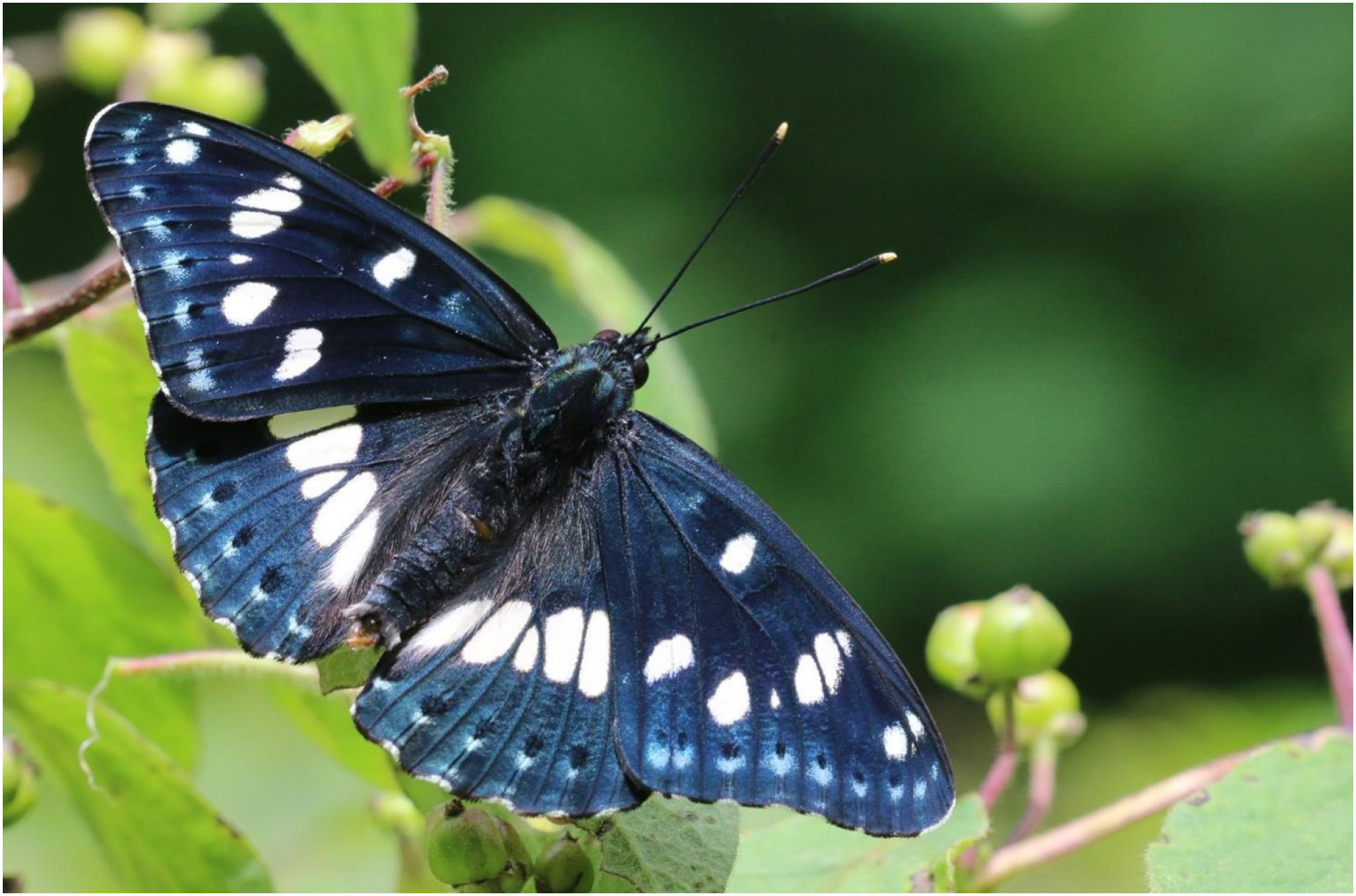
Male Southern White Admiral (*Limenitis reducta*) on its larval host plant, the Fly Honeysuckle (*Lonicera xylosteum*).

## 2 MATERIALS AND METHODS

### 2.1 Study species and study area

**FIGURE 2:**
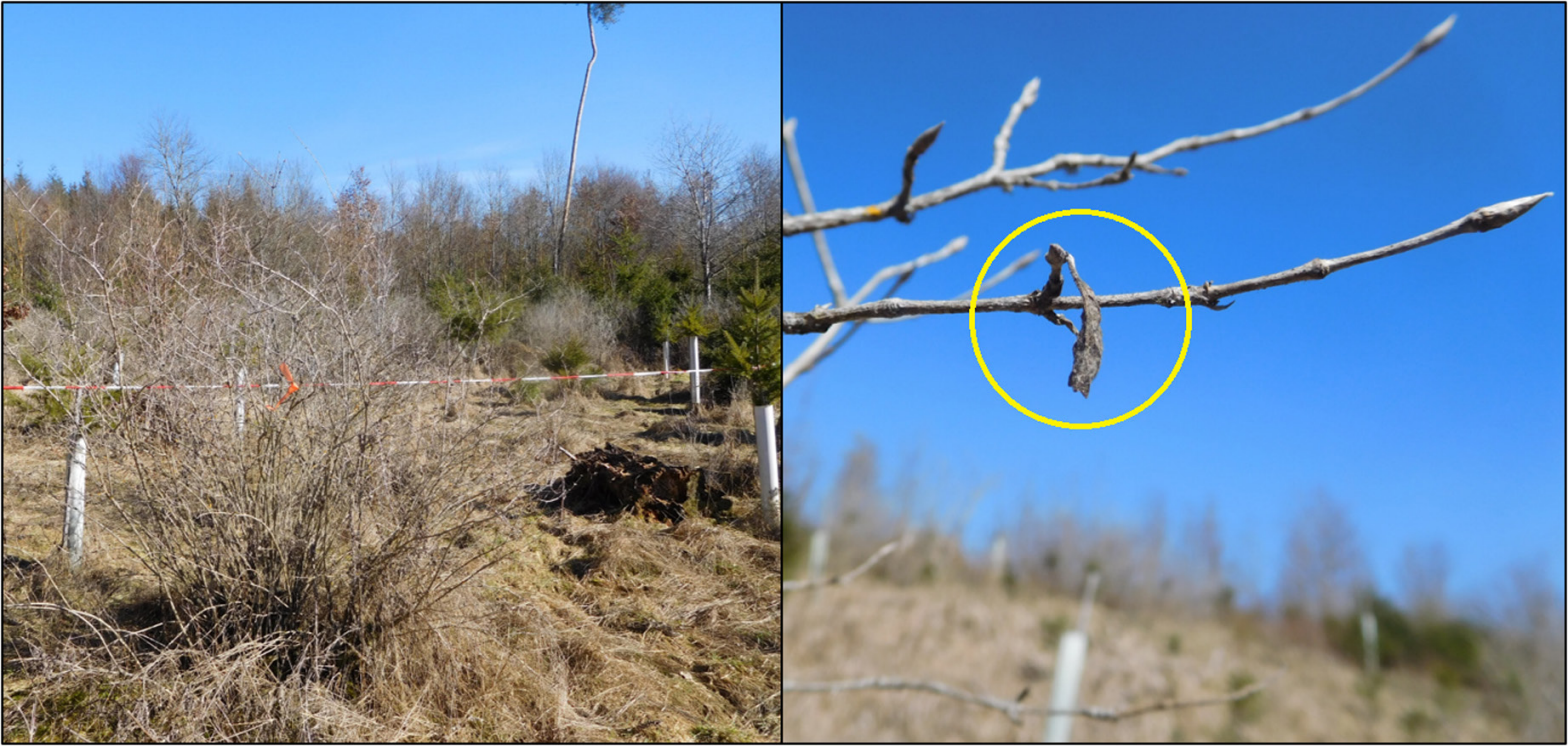
Fly Honeysuckle (*Lonicera xylosteum*) with hibernaculum of the Southern White Admiral (*Limenitis reducta*) on a clear‐cut in the Swabian Jura, February 21, 2021. The barrier tape in the background marks the edge of the study plot (left). Typical hibernaculum of *L. reducta*, attached to the twig of its host plant (yellow circle, right).

We studied a population of the nymphalid butterfly *Limenitis reducta* (Figure 1) in one of its last Central European strongholds in the Swabian Jura. In the study region, *L. reducta* inhabits open forest habitats and clearings. Adults are on the wing from mid‐June to the end of July; however, population densities in Central European habitats are typically low and the detection of imagines can be difficult. Larvae of *L. reducta* are monophagous in this region and only feed on the leaves of sun‐exposed Fly Honeysuckles (*Lonicera xylosteum* L.; Figure 2). These shrubs are typically found on clear‐cuts and on steep slopes and reach maximum heights of up to 3 m. Females of *L. reducta* deposit their eggs solitarily on the host plant. Early in September, larvae start a diapause in the third larval instar and hibernate inside a shelter (“hibernaculum”), which is built from a honeysuckle leaf and which is attached to the twig with a silk thread (Hermann, 2007; Figure 2). The hibernacula can differ in their shape and are typically 0.5–2 cm in size. Ideally, all honeysuckle leafs with the exception of the hibernacula fall off in late autumn/early winter. Consequently, the best time for detecting hibernacula is from December to April. The presence of one hibernaculum does always indicate the presence of one larva immediately after the start of the diapause. A certain percentage of larvae may die during hibernation and an increasing percentage of empty hibernacula may, therefore, be found toward the end of the winter.

The co‐occurring and closely related White Admiral (*Limenitis camilla*) can also be found on *L. xylosteum* bushes and has the same hibernation strategy (Hermann, 2007; Strätling, 2010). However, in the Swabian Jura, *L. camilla* prefers moist, often fully shaded situations. Very rarely, hibernacula of both *Limenitis* species can be found in the same habitat patch (Hermann, 2007). Fortunately, the morphology of their hibernating larvae allows for a clear distinction between the two species. Larvae of *L. reducta* have a grayish color and bear dense crowns of thorns on their back. In contrast, larvae of *L. camilla* are less spiny and more reddish in color (Hermann, 2007).

Our study site was located on a clear‐cut of former Norway spruce (*Picea abies* L.) near Merklingen, NW of Ulm, Southern Germany (48.494°N, 9.788°E, 677 m a.s.l.). It was subdivided into three study plots (A, B, and C), marked with barrier tape. The plots were chosen such that each plot contained about 100 to 150 sun‐exposed bushes of *L. xylosteum*, corresponding to areas of 916 m^2^ (A), 807 m^2^ (B), and 2952 m^2^ (C). The habitat was relatively homogenous within but differed between the plots. Host plant density was high (app. 15 *L. xylosteum* bushes per 100 m^2^) in plots A and B but moderate (app. 4 *L. xylosteum* bushes per 100 m^2^) in plot C. The density of young conifers (*P. abies*, *Pseudotsuga menziesii* Franco) with some shading effect on *L. xylosteum* was highest in plot B.

### 2.2 Data collection

Within a time period of six weeks (February 5, 2021–March 22, 2021), 13 surveyors of different experience (6 experts and 7 novices) participated in a survey for hibernating larvae of *L. reducta*. While novices had no or minimal experience, experts had conducted searches for hibernacula of *L. reducta* at least 10 times prior to our study. The surveyors independently searched for *Limenitis* hibernacula in the study plots. Seven surveyors searched in all three plots, two surveyors searched in two plots, and four surveyors searched in only one plot. Consequently, plots A, B, and C were surveyed by 10, nine, and 10 different surveyors, respectively. All surveyors were allowed to search for a maximum of 2 hr per plot. For most surveyors, this time budget allowed a very thorough search, aiming at finding all hibernacula in a plot. The surveyors conducted their searches, one after the other, without prior information and without seeing each other. Each surveyor marked all detected hibernacula with a colored textile tape. Immediately after a surveyor had finished his/her search, the first author recorded each detected hibernaculum with GPS coordinates (Garmin Oregon 700, spatial precision in the field app. 5–10 m) and a photograph, including the corresponding compass direction. In some cases, he noted additional information facilitating retrieval of the hibernaculum, for example, prominent characteristics of the host plant or the surrounding vegetation. After data collection and before the next surveyor entered the study plot, the first author removed the marking tapes. The first author himself also participated in the survey and was the first surveyor in all plots, thus without prior information about the numbers and positions of the recorded hibernacula. Immediately after the last surveyor had finished, we counted the hibernacula detected by each individual surveyor and matched those with the records of the others. Furthermore, we determined the species of the larvae inside the *Limenitis* hibernacula from their morphology. Three hibernacula disappeared during the study period, that is, the exact positions could be retrieved but the hibernacula were lost or only the silk threads remained. To meet the assumption of population closure, we removed the three hibernacula from our dataset.

### 2.3 Data analysis

Besides the *minimum larva number (MLN)*, which could be directly inferred from our field data, we tested different statistical approaches to estimate the larva population in the study plots.

1. The *removal method*: While the number of new captures decreases non‐linearly from capture occasion to capture occasion, the relationship between the number of new captures (*y*‐variable) and the number of previously captured individuals (*x*‐variable) follows a linear downward trend when the capture probability is constant between capture occasions (cf. Kupfer & Schlüpmann, 2009). Consequently, the *x*‐axis intercept of a linear regression model estimates population size. In our study, different surveyors have worked independently and without prior knowledge about positions and numbers of hibernacula. The order in which the surveyors conducted their searches can thus be randomly reshuffled during the analysis, enabling the calculation of confidence intervals. We permutated our datasets for each plot 10,000 times, that is, we randomly changed the surveyor order and, therefore, the positions of zeros and ones within the capture histories of individual hibernacula. For each permutated dataset, we then calculated a linear regression model with the records of the *n*th surveyor as dependent and the number of previously detected hibernacula as explanatory variables (Leslie & Davis, 1939; Rodriguez de Rivera & McCrea, 2021). Finally, we determined the median estimated population size and 95% confidence intervals from the 10,000 permutations. The datatable and R‐script in the Supplementary Material provide detailed insights into data structure and the analytical procedure.
2. *Full effort CMR models*: We combined the data from the three plots and from all surveyors (= full personnel effort) and estimated detection probabilities across study plots with the Huggins model for closed populations in RMark (Huggins, 1989; Laake, 2013). First, we modeled the probabilities of capture (p) and recapture (c) as shared, time‐dependent parameters, allowing the estimation of surveyor‐specific detection probabilities. Plot‐ID was included as grouping factor such that estimates of caterpillar abundance in each plot could be derived from the model. Second, we included surveyor experience as a occasion‐specific covariate and modeled p and c dependent on the level of surveyor experience.
3. *Reduced effort approaches:* As experts on butterflies are limited, a reduced number of recorders may be more realistic to conduct a long‐term population monitoring or a comparative population study. Therefore, we tested CMR approaches with less personnel and restricted our analysis to the expert dataset. We analyzed the data with the Huggins model and derived estimates of hibernacula abundance in the three plots under all possible combinations of two to six expert surveyors. We modeled p and c as shared and constant parameters when the dataset consisted of only two survey rounds and as shared and time‐dependent parameters otherwise.

Furthermore, we used the mean detection probability of experts and the surveyor‐specific detection probabilities as scaling factors to estimate the hibernacula population from the detections of one expert surveyor only.

As a measure of accuracy, we calculated for each population estimate ${\hat{N}}_{i}$ the relative error compared with the estimated population size, ${\hat{N}}_{f}$, from the *full effort CMR* model: $\text{relative error}=\frac{\;\sqrt{{\left({\hat{N}}_{i}-{\hat{N}}_{f}\right)}^{2}}}{{\hat{N}}_{f}}$. For estimates from *reduced effort CMR* models, we used the relative width of the 95% confidence interval as a measure of precision: $\text{relative width}\;{\mathrm{CI}}_{95\%}=\frac{\text{upper limit}\;{\mathrm{CI}}_{95\%}\left({\hat{N}}_{i}\right)-\text{lower limit}\;{\mathrm{CI}}_{95\%}\left({\hat{N}}_{i}\right)\;}{{\hat{N}}_{i}}$.

To test whether the assumption of equal detectability among hibernacula was met, we compared observed detection frequencies to expected frequencies under equal detection. For that, we simulated 10,000 detection histories for each individual hibernaculum, assuming the population estimate from the *full effort CMR* model as true population size and conducting random draws from the population according to the number of detected hibernacula by the individual surveyors. All statistical analyses were conducted within R, version 3.6.0 (R Core Team, 2019).

## 3 RESULTS

**FIGURE 3:**
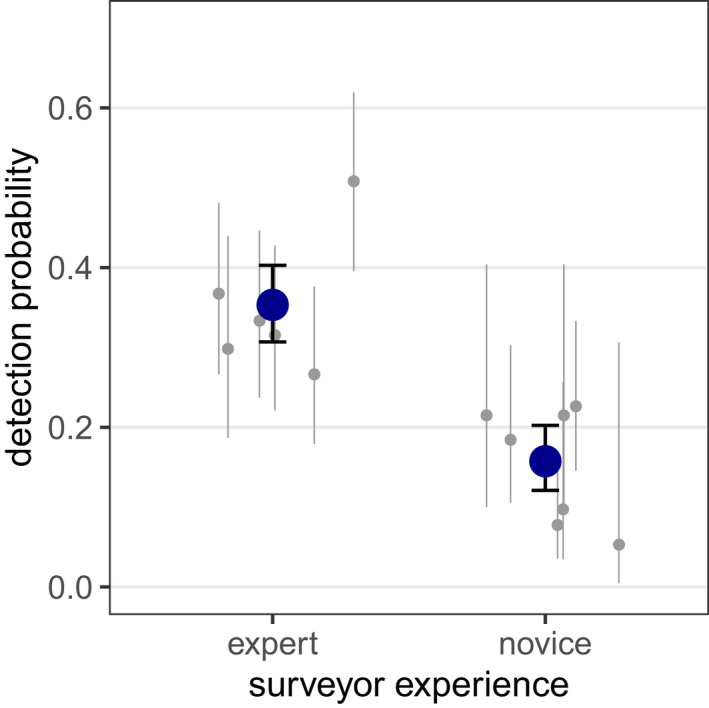
Detection probabilities of *L. reducta* hibernacula for surveyors of different experience. Jittered gray dots represent estimates for individual surveyors, accompanied by 95% confidence intervals. Blue dots and error bars represent group‐specific mean detection probabilities and 95% confidence intervals for experts and novices.

**FIGURE 4:**
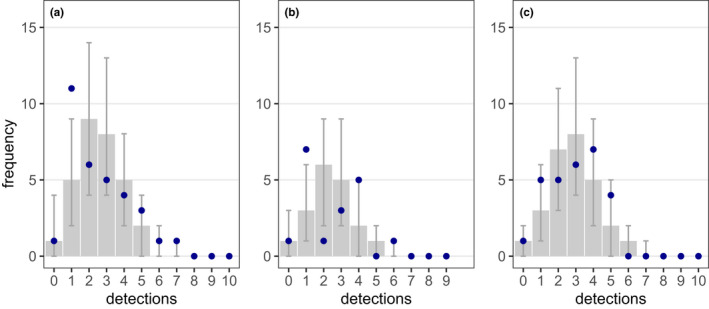
Frequency of hibernacula detections in the three plots (a, b, c; blue dots) and in 10,000 simulations with equal detection probability among hibernacula (gray bars, representing the median; error bars, representing 95% confidence intervals). Ideally, observed frequencies should be identical to simulated detection frequencies. At least most of the observed frequencies are within 95% confidence from the expected frequencies, indicating almost equal detection probabilities among hibernacula. In each plot, estimated hibernacula numbers exceeded the number of observed hibernacula (MLN) by one, meaning that one hibernaculum per plot remained undetected by all surveyors.

In plots A, B, and C, a total of 31, 17, and 27 different hibernacula were detected by 10, nine, and 10 independent surveyors. Estimates of the larval abundance generated under the *removal method* and the *full effort CMR* model did only slightly exceed the counted *minimal larva numbers*. R*emoval* and *full effort CMR* models provided identical population estimates, but the CMR approach had a higher precision, that is, smaller 95% confidence intervals (Table 1). Detection probabilities of novice and expert surveyors differed considerably with a mean detection rate of 16% in novices and 35% in experts. Even the most successful expert could on average not detect more than 51% of the hibernacula in a plot (Figure 3). Comparing observed with expected detection frequencies revealed no severe deviations from the assumption of equal detectability (Figure 4).

**TABLE 1:** Population sizes of *L. reducta* larvae in the study plots, derived from three different approaches: *minimum larva number*, *removal method*, and *full effort CMR*. We present estimates accompanied by 95% confidence intervals.

Plot	Minimum larva number	Removal method	Full effort CMR
A	31	32 (28–47)	32 (31–37)
B	17	18 (14–38)	18 (17–22)
C	27	28 (24–36)	28 (27–33)

**FIGURE 5:**
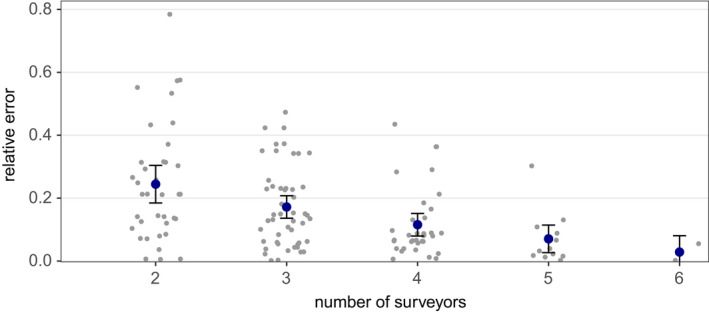
Relative error of population estimates under *reduced effort CMR* approaches with two to six expert surveyors. Blue dots show mean values ±1.96 SE. Jittered dots represent the accuracy of individual population estimates. The relative error of an individual estimate ${\hat{N}}_{i}$ was calculated as $\frac{\;\sqrt{{\left({\hat{N}}_{i}-{\hat{N}}_{f}\right)}^{2}}}{{\hat{N}}_{f}}$ with ${\hat{N}}_{f}$ being the population estimate from *full effort CMR*.

**FIGURE 6:**
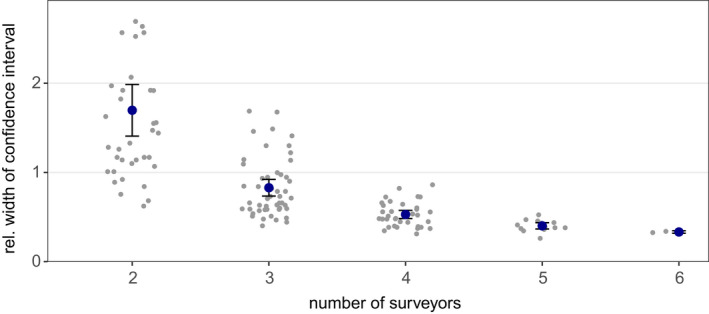
Relative width of the 95% confidence intervals of population estimates derived under *reduced effort CMR* approaches with two to six expert surveyors. Blue dots show mean values ±1.96 SE. Jittered gray dots represent the precision of individual population estimates. The relative width of an individual 95% confidence interval was calculated by dividing the width of the interval by the respective population estimate.

**FIGURE 7:**
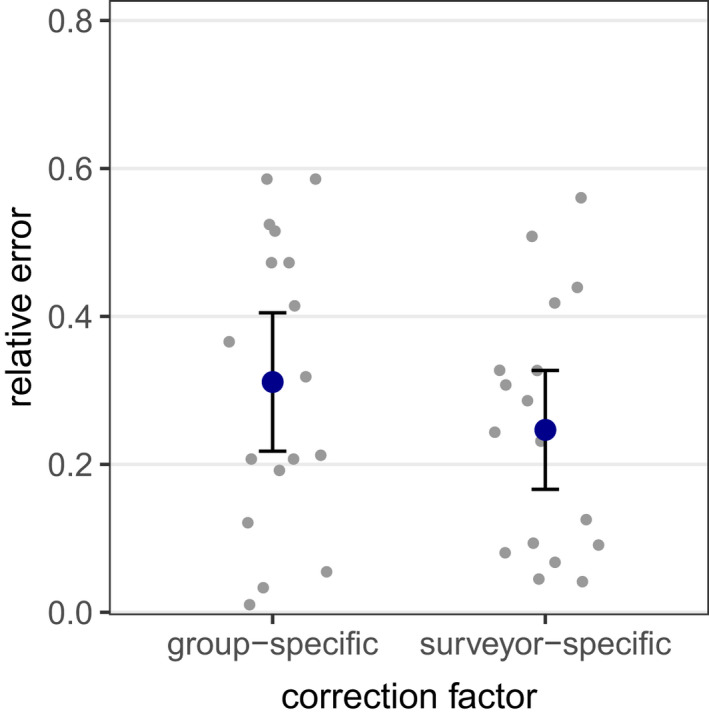
Relative error of population estimates under *reduced effort approaches* with one expert surveyor only. Blue dots show mean values ±1.96 SE. Jittered gray dots represent the accuracy of individual population estimates, derived from the surveyor's record and the group‐specific detection probability of experts (left) / the surveyor‐specific detection probability (right). Detection probabilities were estimated with the *full effort CMR* model.

In 136 out of 140 *reduced effort CMR* models, population size could be reliably estimated. Within these 136 models, we observed a gradual increase in accuracy and precision with increasing surveyor number (Figures 5 and 6). With three expert surveyors, population estimates had a mean error of 17% and a relative confidence interval width of 83%. Under the survey regime with four experts, mean error and confidence interval width further improved to less than 12% and 53%, respectively. On average, with the *reduced effort CMR* approaches, sizes of the hibernacula populations were slightly underestimated. Population estimates derived from the records of a single expert had a similar accuracy as CMR estimates from two surveyors, irrespective of whether the surveyor‐specific or the group‐specific mean detection rates were used as scaling factors (Figure 7).

Among the 75 hibernacula observed within the three study plots, 39 were assigned to *L. reducta* while two hibernacula were from *L. camilla*. Thirty‐four hibernacula were without larva, that is, the caterpillar may have been predated during the 6 months from the start of hibernation to the end of our study. Taking full and empty hibernacula and the proportions of both species into account, estimated densities of *L. reducta* larvae ranged between 0.95 and 3.03 per 100 m^2^.

## 4 DISCUSSION

Applying different approaches of population estimation to larvae of *Limenitis reducta*, we showed that the removal and the CMR method with multiple surveyors can be powerful tools for estimating abundance of overwintering pre‐imaginal butterfly stages.

### 4.1 Validity of the approach

Two fundamental assumptions of removal and closed population CMR models were certainly met by our study system, that is, the absence of births and the absence of immigration/emigration. Mortality during hibernation may have little effect on the population estimates because the hibernacula usually remain, even if the caterpillars inside are predated. Therefore, the period to collect comparable abundance data, that is, from complete leaf shedding to leaf budding, is long (>4 months) compared with the short flight period of adults. Using pre‐imaginal surveys, a high number of different habitat patches can thus be studied within the same season.

We observed almost equal detectability among hibernacula in our experiment, indicating that the assumption of random mixing of individuals between consecutive capture occasions can indeed be relaxed if each capture occasion is conducted by another surveyor. Consequently, mobility of the organism under study is no longer a prerequisite for using removal or closed population CMR techniques. Slight differences in the detectability of hibernacula may have resulted from variable hibernacula size, differences in the height and position at the host plant as well as varying accessibilities of individual host plants.

### 4.2 Detection probability and abundance of *L. reducta* hibernacula

The chance of an individual surveyor to detect a hibernaculum varied strongly with surveyor experience and was more than twofold in experts (35%) compared with novices (16%). Even though detection was imperfect for an individual surveyor, our estimates indicate that nine/ten surveyors together found nearly all available hibernacula in a plot. Besides the surveyor's experience, the visual capacity and concentration, the search pattern, and the light conditions might have affected the detection probability of an individual observer. Even very experienced surveyors could detect only about half of the hibernacula in a plot and taking detection probability into account is therefore crucial when estimates of total abundance are required.

The densities of *L. reducta* caterpillars are of special interest for species conservation purposes. We estimated one to three individuals per 100 m^2^ patch area in one of the species' last strongholds in Central Europe. This corresponds to 13–24 caterpillars per 100 suitable host plants. The hibernacula density per area was highest when host plant density was high and no shading occurred (plot A). The highest hibernacula density per host plant was reached under a lower host plant density (plot C). Despite a high host plant density on plot B, the hibernacula density was lower compared with plot A, most likely because of the shading effects of some larger trees. Based on our data gathered from other nearby study areas comprising more than 10 ha in total, we conclude that the values estimated in this study are above the average caterpillar densities across years and habitat patches in the Swabian Jura. Densities in Mediterranean regions, where the species is more abundant, unfortunately have so far not been determined.

### 4.3 Best sampling scheme for *L. reducta*

We compared the population estimates from sampling schemes with different personnel effort and found that, with regard to the precision of estimated population sizes, CMR is superior to the removal method under the same personnel effort. The accuracy and the precision of estimates from the multi‐surveyor CMR approach asymptotically increased with increasing sampling effort, that is, a rising number of surveyors.

Local butterfly populations can fluctuate substantially between years, with coefficients of variation (CV) between 0.5 and 1.4 being common for different species and in different habitats (e.g., Franzén et al., 2013; Nowicki, 2017; Oliver et al., 2010). Our results show that a multi‐surveyor CMR scheme aiming at detecting such typical abundance fluctuations with 95% confidence in a population of hibernating *L. reducta* requires three to four experts. The required number of surveyors may be different for other species and has to be determined.

One option to further reduce the personnel effort required for population estimation may be to extrapolate total hibernacula abundance from the records of one individual expert only. When the surveyor‐specific detection probability has been determined, it can be used as a scaling factor for the counts of a single expert surveyor. Then, a similar accuracy as under the multi‐surveyor CMR approach with two surveyors is reached. When the observer‐specific detection rate is unknown, the mean detection rate for experts can be used. Generally, sampling of one surveyor only comes with the uncertainty that detection probabilities may vary to an unknown extent between different habitats, which cannot be accounted for without repeated sampling. Another possibility to reduce the number of experts needed may be to substitute multiple surveyors by one surveyor sampling repeatedly along multiple random walk pathways. The random walk pathways could be computer‐simulated before the campaign and uploaded to a mobile mapping device. However, precision of GPS is not sufficient to apply simulated random walk pathways in typical *L. reducta* habitats. A precision of approximately 1 m would be necessary to differentiate reliably between neighboring host plants but this precision is difficult to obtain, even when a DGPS receiver might be used. Furthermore, the experience from our study shows that, sometimes, different surveyors can detect and can miss some of the hibernacula on the same host plant. When visiting the same plant multiple times, an individual surveyor could be biased toward a specific hibernaculum that he/she remembers from the previous detection. Thus, when high‐accuracy population estimates are desired, multi‐surveyor sampling should be conducted whenever possible.

### 4.4 Strengths and limitations of the approach and its applicability to other species

Despite a rising awareness in many disciplines, imperfect detection is still often ignored in entomology and plant surveys, possibly introducing some bias into research outcomes (Kellner & Swihart, 2014). Chen et al. (2013) suggest to estimate detection probability in distribution studies of plants and other sessile organisms using replicate surveys and site‐occupancy models. Single‐survey occupancy models can be fitted when informative covariates are available (Lele et al., 2012). While occupancy models and CMR share common principles, they have different purposes. Taking detection probability into account, occupancy models estimate the probability of true presence of a species at a site (e.g., Korner‐Nievergelt et al., 2015) whereas CMR models estimate abundance conditional on species presence. Both methodological toolsets provide uncertainty measures. This is important, because analyses of long‐term butterfly monitoring data have shown that population parameters such as growth rate, carrying capacity, and temporal population stability cannot be properly modeled when the measurement error is unknown (Hovestadt & Nowicki, 2008; Nowicki, 2017). For butterflies, recording of pre‐imaginal stages and multi‐surveyor CMR techniques combine ecological and field methodological merits with statistical rigor, in particular the estimation of absolute instead of relative population sizes, and the quantification of measurement errors. The multi‐surveyor CMR approach reliably estimates population sizes of immobile butterfly larvae, and it can be easily applied and can be fitted to a wide range of sessile organisms, for example, some plants, fungi, or corals.

Regarding the quantitative monitoring of butterflies, the proposed approach is particularly suitable for species with conspicuous pre‐imaginal stages, for example, *Limenitis camilla*, *Satyrium ilicis*, *S. spini*, *S. acaciae*, *Aporia crataegi*, *Euphydryas aurinia*, *Melitaea aurelia*, and *M. cinxia*. Multi‐surveyor CMR may be limited by very low detection probabilities in species with cryptic larvae, for example, most Satyridae, or when detection probability is per se unequal among individuals. For example, larval stages of *Apatura* spp. can be easily detectable in lower but inaccessible in upper tree layers.

The analysis of CMR data is relatively straightforward with modern software packages, for example, MARK or RMark (Laake, 2013; White & Burnham, 1999). But, the application of multi‐surveyor CMR requires high effort in the field. Three to four experts can estimate the abundance of overwintering *L. reducta* in a habitat patch with 400 host plants in approximately one working day. The larval habitat of a *L. reducta* population typically contains several thousand host plants, distributed over different habitat patches. Estimating total population size using multi‐surveyor CMR during hibernation may, therefore, be of similar effort to estimating the population size of imagines in a classical CMR study. However, the time period when data collection is possible is much shorter for the population of adults than for the hibernating larvae.

It turned out that retrieving all hibernacula without in‐field markings is challenging but can be well accomplished when a combination of GPS for medium‐scale and photographs for fine‐scale redetection is used. Following well‐defined standards for the imaging process, for example, the same compass direction and the same camera position relative to the hibernaculum for all photographs, matching of the records may potentially become feasible without in‐field retrieval. However, different kinds of obstacles such as trees or neighboring honeysuckle bushes, which might reduce a clear view to the hibernacula, could complicate the application of such standards. The high effort required for multi‐surveyor CMR of pre‐imaginal stages may limit its benefit for common species or those that are easily detectable as adults. However, for the quantitative monitoring of elusive species with a high species conservation relevance, such as *L. reducta* in Central Europe, the use of multi‐surveyor CMR with multiple experts is justified and has a strong effort‐to‐benefit ratio.

For using the results for population viability analyses, a clear distinction between pre‐imaginal and adult abundances is crucial because the difference can be several orders of magnitude, depending on the considered pre‐imaginal stage. Our population estimates refer to the larval abundance right after the start of hibernation. Overwinter mortality in butterflies is typically high, for example, 51% and 38% in larvae of *Lasiommata maera* and *Lopinga achine* (Gotthard et al., 1999), 50%–75% in *Euphydryas phaeton* (Abarca et al., 2019), and may strongly compromise the conclusions that can be derived from counts of eggs or young larvae concerning adult population sizes. It is one of the great advantages of our approach to estimate overwinter mortality as a part of the survey. We have observed 47% empty hibernacula of *Limenitis* on the last day of our survey in late March, approximately four weeks before the caterpillars leave their hibernacula. Three out of 78 hibernacula (4%) disappeared during the six weeks of our study. Our field observations at >500 hibernacula of *L. reducta* suggest that the percentage of hibernacula disappearing completely from the beginning to the end of hibernation varies between only 1% and 7%. Hence, the percentage of empty hibernacula in March seems to be a reasonable approximation of overwinter mortality, although it cannot account for some larvae not leaving their hibernacula in spring despite having appeared healthy a few weeks before. Altogether, our records suggest a mortality of approximately 92% from the beginning of hibernation until the emergence of adult *L. reducta* (unpublished data). Multiplying the estimated hibernacula abundances with the survival rates of later life stages can provide estimates of recruitment. In our case, the populations of 18–32 overwintering larvae would translate into an estimated recruitment of 1–3 adult *L. reducta* per plot, corresponding to 7–24 adults per hectare larval habitat. Larval survival rates may vary substantially between years and habitat patches, and we acknowledge that estimating adult population sizes from larval abundances should be treated with caution. This is especially true because different larval habitat patches may be connected through dispersing adults and thus belong to the same spatially structured population.

## 5 CONCLUSION

We conclude that pseudo‐removal and in particular multi‐surveyor CMR are very useful techniques for estimating the abundance of pre‐imaginal butterfly stages. The approaches have a high potential for quantitative studies of other elusive sessile organisms or specific sessile life stages. Despite some limitations concerning the interpretation of abundance data from pre‐imaginal stages in the context of population viability, the recording of eggs, larvae, and pupae provides several benefits compared with the recording of adult butterflies. The multi‐surveyor CMR approach allows the estimation of absolute population sizes together with confidence intervals and can increase the methodological transparency of butterfly sampling schemes. Therefore, we strongly encourage the quantitative study of pre‐imaginal butterfly stages and the use of multi‐surveyor CMR techniques.

## AUTHOR CONTRIBUTIONS

**Heiko Hinneberg:** Conceptualization (lead); formal analysis (lead); investigation (equal); methodology (lead); project administration (equal); writing – original draft (lead); writing – review and editing (equal). **Jörg Döring:** Conceptualization (supporting); investigation (equal). **Gabriel Hermann:** Conceptualization (supporting); investigation (equal); writing – original draft (supporting); writing – review and editing (supporting). **Gregor Markl:** Investigation (equal); writing – original draft (supporting). **Jennifer Theobald:** Investigation (equal); writing – original draft (supporting); writing – review and editing (supporting). **Ines Aust:** Investigation (equal). **Thomas Bamann:** Investigation (equal). **Ralf Bertscheit:** Investigation (equal). **Daniela Budach:** Investigation (equal). **Jana Niedermayer:** Investigation (equal). **Alicia Rissi:** Investigation (equal). **Thomas K. Gottschalk:** Conceptualization (supporting); funding acquisition (lead); investigation (equal); project administration (equal); supervision (lead); writing – original draft (supporting); writing – review and editing (equal).

## CONFLICT OF INTEREST

We have no conflicts of interest to declare.

## Supporting information


Appendix S1

## Data Availability

Data and R‐scripts are publicly available: 10.5061/dryad.s7h44j195.
